# Developing a knowledge base to support the annotation of ultrasound images of ectopic pregnancy

**DOI:** 10.1186/s13326-017-0117-1

**Published:** 2017-01-31

**Authors:** Ferdinand Dhombres, Paul Maurice, Stéphanie Friszer, Lucie Guilbaud, Nathalie Lelong, Babak Khoshnood, Jean Charlet, Nicolas Perrot, Eric Jauniaux, Davor Jurkovic, Jean-Marie Jouannic

**Affiliations:** 1 UPMC Medical Faculty (Paris 6), Department of Fetal Medicine in Armand Trousseau Hospital (APHP), INSERM U1142 (LIMICS), 26 Avenue du Dr Arnold Netter, 75012 Paris, UE France; 2 INSERM U1153 (Obstetrical, Perinatal and Pediatric Epidemiology Research Team, Center for Biostatistics and Epidemiology), Maternité Port Royal, 53 Avenue de l’Observatoire, 75014 Paris, UE France; 3 APHP DSI, INSERM U1142 (LIMICS), 15, rue de l’École de Médecine, 75006 Paris, UE France; 4 Pyramides Medical Imaging Center, 13 av. de l’Opéra, 75001 Paris, UE France; 5 University College Hospital (UCLH) Department of Obstetrics and Gynaecology, Academic Department of Obstetrics and Gynaecology, University College London (UCL) Institute for Women’s Health, 86-96 Chenies Mews, London, WC1E 6HX UE UK; 6 Department of Obstetrics and Gynaecology, Gynaecology Diagnostic and Outpatient Treatment Unit, University College Hospital (UCLH), 235 Euston Road, London, NW1 2BU UE UK

**Keywords:** Application ontology, Knowledge base, Ectopic pregnancy

## Abstract

**Background:**

Ectopic pregnancy is a frequent early complication of pregnancy associated with significant rates of morbidly and mortality. The positive diagnosis of this condition is established through transvaginal ultrasound scanning. The timing of diagnosis depends on the operator expertise in identifying the signs of ectopic pregnancy, which varies dramatically among medical staff with heterogeneous training. Developing decision support systems in this context is expected to improve the identification of these signs and subsequently improve the quality of care. In this article, we present a new knowledge base for ectopic pregnancy, and we demonstrate its use on the annotation of clinical images.

**Results:**

The knowledge base is supported by an application ontology, which provides the taxonomy, the vocabulary and definitions for 24 types and 81 signs of ectopic pregnancy, 484 anatomical structures and 32 technical elements for image acquisition. The knowledge base provides a sign-centric model of the domain, with the relations of signs to ectopic pregnancy types, anatomical structures and the technical elements. The evaluation of the ontology and knowledge base demonstrated a positive feedback from a panel of 17 medical users. Leveraging these semantic resources, we developed an application for the annotation of ultrasound images. Using this application, 6 operators achieved a precision of 0.83 for the identification of signs in 208 ultrasound images corresponding to 35 clinical cases of ectopic pregnancy.

**Conclusions:**

We developed a new ectopic pregnancy knowledge base for the annotation of ultrasound images. The use of this knowledge base for the annotation of ultrasound images of ectopic pregnancy showed promising results from the perspective of clinical decision support system development. Other gynecological disorders and fetal anomalies may benefit from our approach.

## Background

### Ectopic pregnancy is a common early pregnancy complication

Ectopic pregnancy occurs in 1 to 2% of pregnancies in developed countries and is defined by the implantation of a gestational sac outside the endometrial cavity of the uterus [1, 2]. The direct mortality rate from ectopic pregnancy is estimated to be 16.9 per 100,000 ectopic pregnancies [2], and is responsible for 4 to 10% of pregnancy-related deaths around the world [3]. Fallopian tubes are the most common site for ectopics to implant (tubal ectopics) with about 95% of ectopic pregnancies located there. For the rest, the implantation occurs within the uterine wall, but outside the endometrial cavity. Non-tubal ectopics are more difficult to diagnose than tubal ectopics and are associated with a higher mortality and morbidity [4]. Delayed diagnosis is the main factor for ectopic pregnancy associated with maternal death [2] and also affects the success rate of future pregnancies [5].

### Ectopic pregnancy diagnosis relies on ultrasound expertise

The positive diagnosis of ectopic pregnancy is established through ultrasound scanning. More specifically, transvaginal scanning has been demonstrated to be superior to transabdominal ultrasound [4]. Consistent with continuous improvement in imaging quality and expertise, it has been recently suggested that a skilled operator could achieve a definite diagnosis at the very first scan [6]. However, most hospitals still rely on a heterogeneous staff to manage patients at risk for ectopic pregnancy, including emergency physicians, sonographers, radiologists and/or doctors in training [2, 7], with different levels of training and expertise. Thus, three or more visits are needed for 50% of these patients [8].

### A shared representation for ectopic pregnancy imaging

**Fig. 1:**
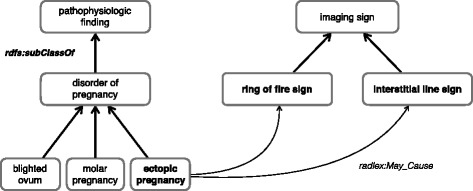
Graphical view of the "ectopic pregnancy" concept from the Radiology Lexicon (RadLex, version 3.13.1), with a full expansion of the concepts neighborhood for "ectopic pregnancy"[RadLex:RID4942]

Existing repositories of medical terminologies and ontologies, namely the Open Biomedical Ontologies (OBO) Foundry [9], the National Center for Biomedical Ontology (NCBO) BioPortal [10] and the Unified Medical Language System (UMLS) Metathesaurus [11] do not include a comprehensive set of resources to represent ultrasound signs. None of the resources reviewed in a recent survey of biomedical imaging ontologies was suitable for ectopic pregnancy [12]. This domain involves concepts from various medical domains, namely medical imaging, human anatomy and obstetrics/gynecology (OB/GYN). While existing standard terminologies may support a formal and shared representation for parts of our domain, as do the Foundational Model of Anatomy (FMA) [13] and the Radiology Lexicon (RadLex) [14], none of them provides the appropriate granularity for ectopic pregnancy imaging. More precisely, RadLex supports the representation of signs from various imaging modalities (including 50 ultrasound imaging signs [15]), as well as their relations to various medical conditions (including ectopic pregnancy), which makes it the best resource for our domain. However, RadLex is insufficient, because there are no subclasses for “ectopic pregnancy” [RadLex:RID4942] and RadLex only provides two related signs (“ring of fire sign” [RadLex:RID35495] and “interstitial line sign” [RadLex:RID35308]), as illustrated in Fig. 1.

### Objectives

In this article, we present a new ectopic pregnancy knowledge base and its application to ultrasound image annotation. In this knowledge base, the signs of ectopic pregnancy are linked to specific types of ectopic pregnancy, the anatomical structures involved and the technical elements of imaging. We also developed an ontology to provide the vocabulary used in the knowledge base, as well as an application for annotating ultrasound images, which leverages the knowledge base. We demonstrate the use of the knowledge base on the annotation of clinical images.

## Methods

**Fig. 2:**
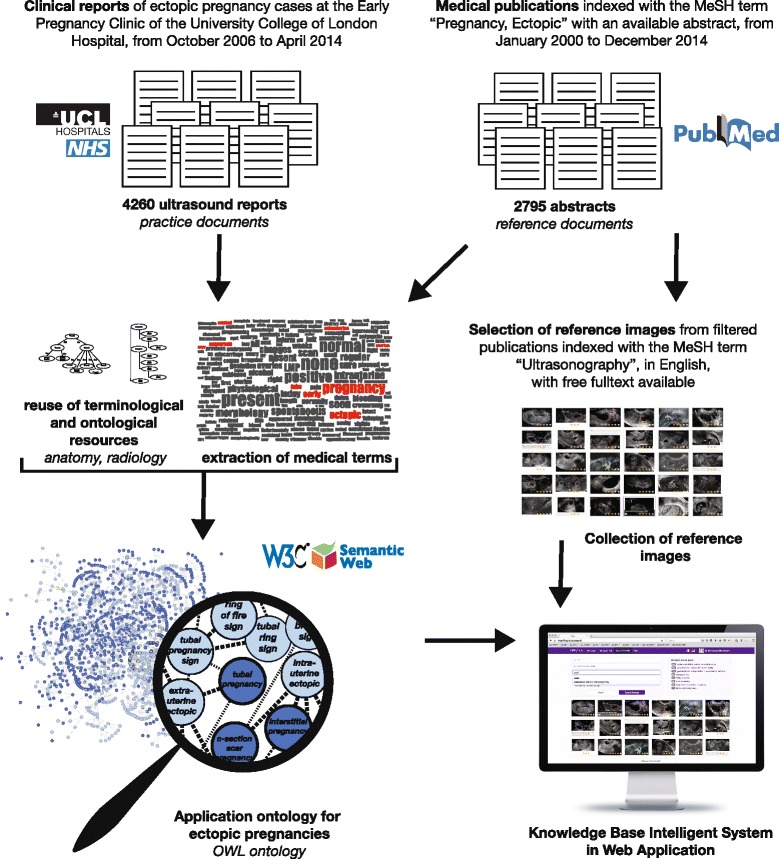
Overview of the design of the knowledge system for ectopic pregnancy: ontology design, reference image collection and application for image annotation

In this section, we describe our approach to developing a knowledge base for ectopic pregnancy imaging. We start by describing the underlying ontology. We present the knowledge base. Finally, we describe the application developed to support the annotation of ectopic pregnancy ultrasound images. The overview of the ontology and knowledge base development is presented in Fig. 2.

### Ontology development

To build the ectopic pregnancy ontology (EPO), we acquired concepts from a medical corpus. We also reused concepts from existing terminologies. We organized these concepts into hierarchies.

### Acquiring concepts from text

We extracted terms from a medical corpus and organized them into concepts.

### Extracting terms from a medical corpus

In order to cover the terms for the features to be annotated on EP images (i.e., types of ectopic pregnancy image, imaging signs, anatomical locations and technical elements for ultrasound image acquisition), we used Natural Language Processing (NLP) techniques [16] to extract and select medical terms from a collection of medical texts from two sources, namely the medical literature and de-identified reports of ultrasound examinations. More specifically, we searched PubMed for all medical publications indexed with the MeSH term "Pregnancy, Ectopic" from January 2000 to December 2014 for which an abstract was available, resulting in a collection of 2795 abstracts. Additionally, we extracted 4260 de-identified ultrasound reports form the Early Pregnancy Clinic database at the University College London Hospital (UCLH), restricted to ectopic pregnancy cases from October 2006 to April 2014. The lexico-syntactic analysis of these texts was performed using the part-of-speech tagger TreeTagger [17] and the term extractor YaTeA (http://search.cpan.org/dist/Lingua-YaTeA/). A total of 40,237 single/multi-word candidate terms were extracted.

### Organizing extracted terms into concepts

The appropriate vocabulary for ectopic pregnancy was developed from these candidate terms, using the platform for ontology development from text Terminae/DAFOE [16]. Two experts reviewed and selected candidate terms, and defined the relevant concepts for ectopic pregnancy image description. The experts followed general principles for ontology design (clarity, coherence, extensibility, minimal encoding bias, minimal ontological commitment) [18–21].

### Acquiring concepts from existing terminologies

Whenever possible, the experts reused elements from existing terminologies, following previously described methods [22, 23]. For example, fine-grained concepts for the description of the pelvic anatomy in the FMA (e.g., the uterus [FMAID:17558] and all its parts) were added to the ontology.

### Organizing concepts into hierarchies

We organized the resulting concepts into a subsumption hierarchy and we added annotations and logical definitions to these concepts.

### Organizing concepts into a subsumption hierarchy

We used a core ontology for the medical domain developed in our academic center (ontoMénélas) to support the interoperability with other resources in our organization [24–29]. The subsumption hierarchy (i.e., *is-a* or *subClassOf* relations) was developed in a top-down approach [19, 30] leveraging expert knowledge in medical imaging and OB/GYN, and by reusing existing resources. In particular, we reused some of the subsumption relations from the FMA (among the FMA concepts that were added to the ontology) as previously described by the RadLex group [31].

### Annotations

All concepts for ultrasound signs of ectopic pregnancy were manually annotated. The minimal set of annotations included (i) one English label (ii) one textual definition in English (iii) one PubMed identifier (PMID) for the concepts extracted from the PubMed corpus. Other annotations were optional (e.g., synonyms and French version of the annotations). The mappings of anatomical concepts to FMA concepts were stored as annotations in the ontology. The FMA labels and definitions for these concepts were also added as annotations. All textual annotations were based on SKOS predicates (*prefLabel*, *altLabel*, *definition*) [32]. We used the biomedical ontology editor Protégé version 5 (http://protege.stanford.edu/) for editing the annotations.

### Logical definitions

General concepts for categories of signs were defined in intension as opposed to extension. These concepts correspond to defined classes in the ontology. For example, the concept “*color Doppler sign*” denotes an imaging sign, visible during an ultrasound examination, using the color Doppler mode. Therefore, this concept is formalized with a logical definition leveraging the property “*requiresMode*” and the concept “*color Doppler mode*”. As a result, signs whose definition contains “*requiresMode* some *color Doppler mode*” would automatically be classified as subclasses of “*color Doppler sign*”.

### Implementation in OWL

The ontology was represented using the Web Ontology Language, OWL [33]. The hierarchy was inferred with an OWL-DL reasoner (Hermit 1.3.8), which also checked the consistency of the ontology.

### Knowledge base development

**Fig. 3:**
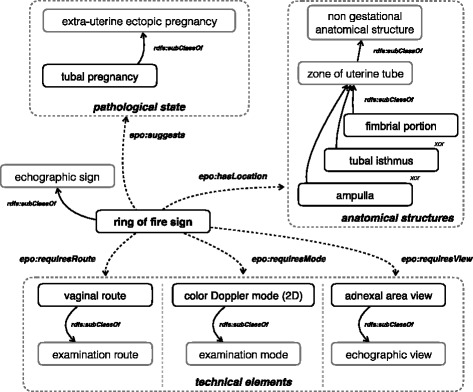
Simplified representation of the sign "*ring of fire*" in the knowledge base

The ontology provides the vocabulary for describing ultrasound images of ectopic pregnancy, which we used for developing a sign-centric knowledge base to represent the relations of each sign to ectopic pregnancy types, anatomical structures and technical elements for the acquisition of ultrasound images. Technical elements include the “*examination route*”, the “*examination mode*”, and the “*echographic view*”. For example, the “*ring of fire sign*” concept is represented in Fig. 3 with its relations to a type of ectopic pregnancy (“*tubal pregnancy*” through the relation “*epo:suggests*”), to anatomical structures (“*ampulla*”, “*tubal isthmus*”, “*frimbrial portion*” through the relation “*epo:hasLocation*”) and to technical elements (“*vaginal route*”, “*color Doppler mode (2D)*”, “*adnexal area view*” through the relations “*epo:requiresRoute*”, “*epo:requiresMode*”, “*epo:requiresView*”, respectively).

This knowledge was asserted at the most general level and propagated through the subsumption hierarchies of the ontology. For example, although the concept “*ring of fire sign*” is not explicitly linked to “*tubal pregnancy sign*” in the knowledge base, this relation can be inferred from “*ring of fire sign epo:suggests tubal pregnancy*” and “*tubal pregnancy sign owl:equivalentClass (epo:suggests* some *tubal pregnancy)*”.

We assessed the domain and scope of the knowledge base using “competency questions”, the answers to which must be represented with relations (asserted or inferred) from the knowledge base [34]. Such questions included “what are the different implantation sites of ectopic pregnancies?”, “what are the imaging signs of cesarean section scar pregnancy?”, “which ultrasound mode is required to depict a ring-of-fire sign?”, and “what are the anatomical structures visible in an adnexal area view?”

Beside the development of the sign-centric knowledge base, we selected from the medical literature ultrasound images of ectopic pregnancies illustrating the signs represented in the knowledge base. We restricted the 2795 PubMed citations used for the text corpus to articles in English, indexed with the MeSH term “Ultrasonography” and for which the article was freely available. One of the authors (PM) selected relevant images from the articles, in which the ultrasound signs were precisely described and illustrated. He annotated the signs in the knowledge base with the PMID of the article. For example, the concept “*ring of fire sign*” is annotated with PMID 18936028 in reference to an article describing this sign [35].

### Application development

We developed an application for the annotation of ultrasound images of ectopic pregnancy. This application leverages both definitional knowledge from the ontology and assertional knowledge from the knowledge base. The main features of this application include:

i) searching for image annotations using terms from the ontology,
ii) suggesting relevant signs based on the knowledge base, and
iii) accessing reference images for a given sign.

The user interface was developed as a Java 7 web application based on open-source elements. The ontology and the knowledge base were stored in an RDF triple store (Apache Jena 3.0). We used queries against a SPARQL endpoint (Apache Fuseki) to access the knowledge base. Simple subsumption reasoning was sufficient to access all the asserted and inferred knowledge from the knowledge base. We established a set of SPARQL rules to suggest the signs, anatomical structures and technical elements associated with a given type of ectopic pregnancy selected by the user. For example, the following SPARQL query retrieves all ectopic pregnancy types having at least one sign from a given set of signs. “${selectedSigns}” is a variable containing the URIs of this set of signs, “${inferredGraph}” is the inferred ontology graph in the triplestore and "${language}" is the language used for label display in the system:

- PREFIX owl:< http://www.w3.org/2002/07/owl #>
- PREFIX rdf:< http://www.w3.org/1999/02/22-rdf-syntax-ns #>
- PREFIX epo:< http://www.semanticweb.org/ontologies/epo.owl #>
- PREFIX skos:< http://www.w3.org/2004/02/skos/core #>
- PREFIX rdfs:< http://www.w3.org/2000/01/rdf-schema #>
- SELECT DISTINCT ?disorder ?disorder_label ?disorder_definition
- FROM ${inferredGraph}
- WHERE {
- VALUES ?sign {${selectedSigns}}
- ?sign skos:hiddenLabel ?sign_id;
- rdfs:subClassOf* epo:OPPIO_0000189 .
- ?disorder skos:prefLabel ?disorder_label;
- rdfs:subClassOf epo:OPPIO_c000016 .
- OPTIONAL {?disorder skos:definition ?disorder_definition .}
- ?sign rdfs:subClassOf* ?restr .
- ?restr owl:onProperty epo:suggests .
- ?restr owl:someValuesFrom/rdfs:subClassOf* ?disorder .
- FILTER(lang(?disorder_label) = "${language}")
- }
- ORDER BY ?disorder_label

The result from this query is a list of ectopic pregnancy types (URI, label and definition) and can be used in subsequent queries to suggest new signs associated with these ectopic pregnancy types.

### Evaluation

We conducted an evaluation of the ontology, the knowledge base and the application. The ontology and the knowledge base were evaluated through a questionnaire and users evaluated the application based on clinical cases.

### Evaluation of the ontology: Does the ontology contain the appropriate vocabulary for ectopic pregnancy ultrasound imaging?

The vocabulary provided by the ontology was presented to a group of potential users with different levels of expertise. After a demonstration of the application followed by a brief hands-on session to search for terms, we collected feedback from each user by anonymous questionnaire. Questions assessed whether the terms for signs, anatomical structures, types of ectopic pregnancy and technical elements were consistent with their clinical practice and if they were able to find the signs they were looking for in the application.

### Evaluation of the knowledge base: Are the suggested signs and images useful?

The signs and images suggested by the knowledge base were assessed by the same panel of users through another questionnaire. We asked users if they learned new signs for some types of ectopic pregnancy and whether the reference images provided were helpful for analyzing ultrasound images. Here we distinguished between junior and senior users, because our intuition was that the juniors are more likely than seasoned physicians to learn from our system.

### Evaluation of the application based on clinical cases

Using our application, users annotated ultrasound images of ectopic pregnancy scans. This study was approved by the ethic committee of the French National College of Obstetrics and Gynecology (No CEROG 2015-GYN-1002). The ultrasound scans (reports and images) were randomly selected from ectopic pregnancy cases managed at the Pyramids Medical Imaging Center in Paris and the Early Pregnancy Unit at UCLH. All personally identifying information was removed from the text of the reports, from the content of the images and from the image metadata. Each observer was assigned a subset of 10 cases for analysis, of which 5 were common to all observers and 5 were specific. For each case, the observers were asked to annotate the images with the application. They were blind to the content of the ultrasound report.

Our motivation for this preliminary evaluation was not so much to assess whether all relevant signs had been annotated, but rather to ensure that the signs suggested by our application were appropriate. In other words, we focus on precision, not recall. Additionally, we evaluated the reproducibility of the annotations among the observers.

### Precision

The gold standard for the presence of signs on each image was derived from the ultrasound reports provided by the specialist centers. We measured the precision of sign annotations provided by the observers (observed signs) against the signs from the gold standard (relevant signs). We used the usual definition for precision in information retrieval [36]:

$$ precision=\frac{\left|\left\{ relevant\  signs\right\}{\displaystyle \cap}\left\{ observed\  signs\right\}\right|}{\left|\left\{ observed\  signs\right\}\right|} $$

### Reproducibility

The measure for assessing the reproducibility of the annotations was the proportion of agreement for categorical assessment across multiple observers [37]. The proportion of agreement ${p}_a$ for a given sign in a given image was the ratio of the number of agreements between the observers (i.e., the number of pairs of observers who agree) for the presence of the sign, over the number $n$ of trials of agreement (i.e., the total number of pairs of observers). For example, considering a group of *x* = 6 observers, of whom 5 observers annotated one of the images with a given sign, the number of trials of agreement is *n* = 1 + 2 + ⋯ + (*x* − 1) = 15, and the number of agreements among the 5 observers is 10. Thus, *p*_*a*_ is 10/15 = 66%. The 95% confidence interval (CI) for ${p}_a$ was calculated from the Standard Error of the proportion: $S E=\sqrt{p_a\left(1-{p}_a\right)/ n}$. Considering a standard normal distribution for *p*_*a*_, the 95% CI is *p*_*a*_ ± 1.96 × *SE*. Statistical computations were performed using R version 3.2 and STATA version 14.

## Results

### Ectopic pregnancy ontology

**Fig. 4:**
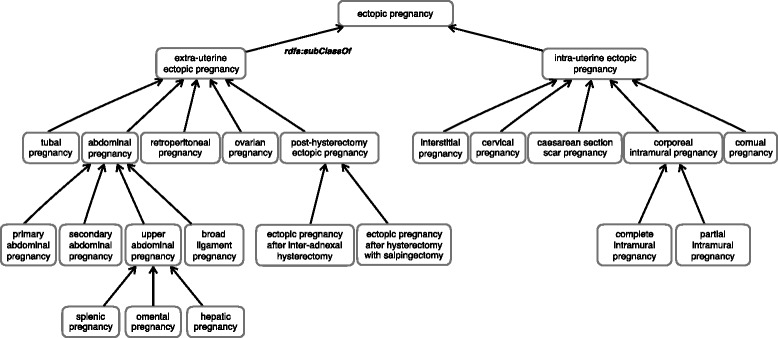
Taxonomy of ectopic pregnancy by implantation sites (view of the Ectopic Pregnancy Ontology)

As of June 2016, the ectopic pregnancy ontology (version 1.1) contains 1388 concepts to describe ectopic pregnancy ultrasound images, organized into several subsumption hierarchies for types of ectopic pregnancies and the signs, anatomical structures and technical elements of imaging associated with ectopic pregnancy. The usual metrics for ontology description are presented in Table 1. There are 24 classes for the types of ectopic pregnancy, as illustrated in Fig. 4. The 90 concepts for ultrasound signs include “*endometrial trilaminar pattern*”, “*tubal ring sign*”, and “*ring of fire sign*”. While most sign concepts are represented as primitive classes, some of them are defined classes. For example, the concept “*color Doppler sign*” is a defined class equivalent to [*rdfs:subClassOf* “*imaging sign*” and *epo:requiresMode* some “*color Doppler mode*”]. In general, we created defined classes for the categories of signs by technical element (e.g., by the examination mode (e.g., “*2D ultrasound sign*”, “*color Doppler sign*”) and by implantation site of ectopics (e.g., “*tubal pregnancy sign*”, “*c-section scar pregnancy sign*”).

**Table 1:** Ectopic Pregnancy Ontology (v1.1) metrics

Class count	1399
Object property count	44
Individual count	0
SubClassOf axioms count	2707
EquivalentClasses axioms count	50
DisjointClasses axioms count	39
AnnotationAssertion axioms count	
- skos:prefLabel	1749
- skos:altLabel	298
- skos:definition	489
- epo:FMAID (FMA class UI)	295

There are 484 concepts for anatomical structures of the female pelvic anatomy (e.g., “*uterus*”, “*uterine tube*”, “*zone of uterine tube*” and “*ampulla*”) and early gestational structures (e.g., “*gestational sac*”, “*trophoblast*”). General anatomical concepts from the FMA were used to seed the hierarchy (e.g., “*organ zone*” and “*non gestational anatomical structure*”). Specialized concepts (e.g., “*gestational sac*”) were added to extend the FMA hierarchy as necessary for our application.

The technical element concepts were organized into three hierarchies for “*examination route*”, “*examination mode*” and “*echographic view*”. There are 3 examination route subclasses (e.g., “*vaginal route*”), 9 examination mode subclasses (e.g., “*color Doppler mode*”), and 17 echographic view subclasses (e.g., “*longitudinal view of the uterus*”).

The asserted subsumption hierarchy of the ontology involved 2707 relations. The domain and range of 44 relations (e.g., “*hasLocation*”, “*suggests*”, “*requiresMode*”) are defined in the ontology. Finally, this ontology includes no individuals, because instances of signs are the actual signs observed on images from a clinical case.

### Knowledge base for image annotation

In the knowledge base, the 81 signs defined in the ontology are related to ectopic pregnancy types, anatomical structures and the three categories of technical elements (the echographic view, the examination mode and the examination route) as illustrated in Fig. 3. There are 169 asserted relations between these signs and the different types of ectopic pregnancy, as some signs can be associated with several types of ectopic pregnancy. Similarly, the signs can be related to multiple anatomical structures (with 239 asserted relations), as well as multiple technical elements (with 356 asserted relations). The asserted knowledge from the sign-centric knowledge base characteristics is summarized in Table 2. After inference in the knowledge base, 618 inferred relations between signs and types of ectopic pregnancy were produced, as well as 1503 inferred relations between signs and technical elements.

**Table 2:** Characteristics of the ectopic pregnancy knowledge base for imaging signs

	Object property	Axioms (n)
Relations		
- Ultrasound Sign ® Ectopic Pregnancy	<epo:suggests>	169
- Ultrasound Sign ® Anatomical Structure	<epo:hasLocation>	239
- Ultrasound Sign ® Technical Element	<epo:requires>	356
Annotations		
- PubMed citations	<epo:PMID>	77
- Image from reference collection	<epo:ImagePath>	98

The signs in the knowledge base are associated with reference images and PubMed citations. One hundred and six articles from 33 medical journals were reviewed for establishing the collection of reference images, resulting in the selection of 80 images depicting relevant ultrasound signs. A total of 77 PMID annotations and 98 image annotations illustrate the signs in the knowledge base.

### Application for ultrasound image annotations

**Fig. 5:**
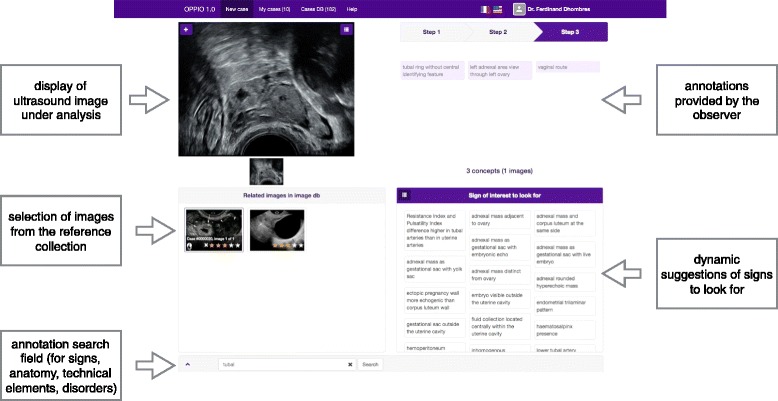
Overview of the interface of the web application for ectopic pregnancy ultrasound image annotations. This graphical user interface was developed using the AngularJS (https://github.com/angular/angular.js) and Bootstrap (https://github.com/twbs/bootstrap)

An overview of the user interface of the application is presented in Fig. 5. The image to annotate is displayed in the top left corner of the screen. The annotation search field at the bottom of the screen supports auto-completion for terms related to ectopic pregnancy types, anatomical locations, technical elements and ultrasound signs. The results are displayed in a sliding panel and the user can select the relevant terms, which are then added as image annotations in the top right corner of the screen as image annotation. As the user selects annotations, the system provides a selection of reference images from the collection in the bottom left corner to illustrate the selected annotations. Finally, in the bottom right corner, the system suggests other signs of interest based on the type of pregnancy, anatomical structure and technical elements selected.

### Evaluation

#### Evaluation of the ontology: Does the ontology contain the appropriate vocabulary for ectopic pregnancy ultrasound imaging?

A total of 17 users (junior and senior OB/GYN practitioners and radiologists, and sonographers from France and the UK) were presented with the application. Their feedback on the terms available in the ontology was generally favorable. More specifically, 100% of the users found the vocabulary for the ectopic pregnancy signs to be consistent with their clinical practice, 94,1% for the anatomical structures and 82,4% for the terms describing technical elements of imaging. Moreover, 82.4% were able to find the signs they were looking for in the application, without further assistance.

### Evaluation of the knowledge base: are the suggested signs and images useful?

Overall, half of the users (52.9%), including all five junior users, learned about new signs associated with ectopic pregnancy types. The reference images suggested by the application were “always” of “often” useful for 14 users (82.4%). One user considered that the suggested images were “sometimes” useful and two users considered the suggested images “rarely” useful. As expected, the usefulness of the application depended on the expertise of the user, with junior users benefitting most.

### Evaluation of the application based on clinical cases

**Fig. 6:**
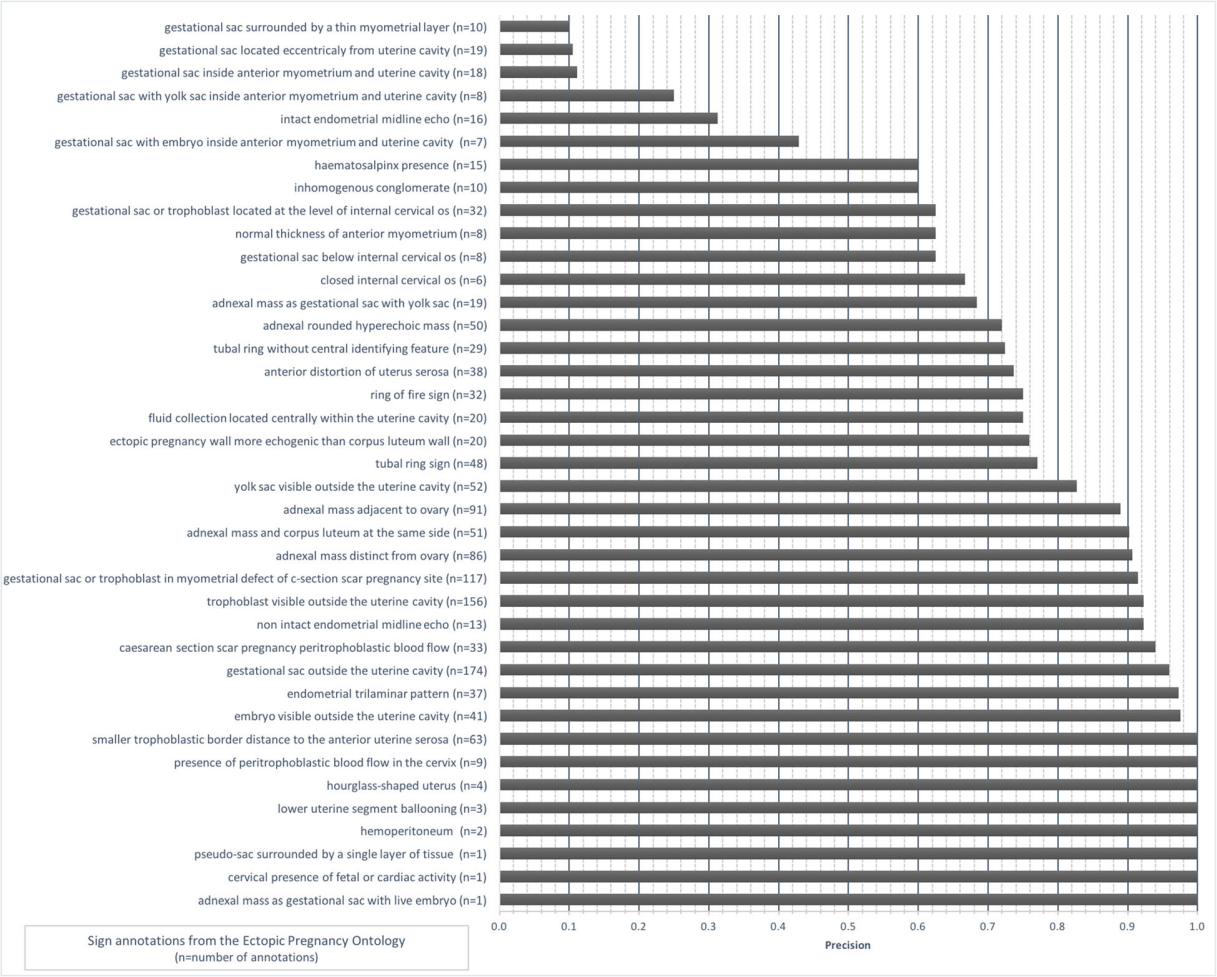
Precision of ectopic pregnancy sign annotations in ultrasound images

Six independent observers, all OB/GYN practitioners with different level of training in ultrasound imaging (three seniors, two senior registrars and one registrar) annotated 206 ultrasound images from 35 clinical cases of ectopic pregnancy (five common cases and five additional cases for each user). The cases are presented in Table 3. The observers provided 1486 annotations with an overall precision of 0.83. The precision for each sign is presented in Fig. 6.

**Table 3:** Types of ectopic pregnancy among the 35 ultrasound cases used for the annotation evaluation

Type of ectopic pregnancy	Cases in common	Other cases
Tubal pregnancy	3 (20 images)	12 (67 images)
Cesarean section scar pregnancy	2 (13 images)	12 (74 images)
Cervical pregnancy	-	4 (24 images)
Interstitial pregnancy	-	2 (8 images)
Total	5 (33 images)	30 (173 images)

For the five common cases, the observers used 46 distinct signs to create 841 annotations. For 783 annotations (covering 26 distinct signs), the annotation was made by at least two observers. The 58 remaining annotations (6.9%) were created by only one of the six observers and involved 20 distinct signs. The total proportion of agreement for the presence of signs in images was 40.35% [38.64%-42.05%]_95%CI_. The reproducibility for each sign annotation is presented in Table 4.

**Table 4:** Proportion of agreement on the presence of ultrasound signs in the 5 common cases of ectopic pregnancy

Sign annotations form the Ectopic Pregnancy Ontology	Agreement (*n*)	Lack of agreement (*n)*	Images (n)	Proportion of agreement (% and [95% CI])
Endometrial trilaminar pattern	48	12	4	80.00 [69.88 - 90.12]
Adnexal mass distinct from ovary	143	37	12	79.44 [73.54 - 85.34]
Embryo visible outside the uterine cavity	45	15	4	75.00 [64.04 - 85.96]
Gestational sac outside the uterine cavity	226	104	22	68.48 [63.47 - 73.49]
Adnexal mass adjacent to ovary	122	58	12	67.78 [60.95 - 74.61]
Gestational sac or trophoblast in a myometrial defect in previous caesarean section scar pregnancy site	119	76	13	61.03 [54.18 - 67.88]
Adnexal mass and corpus luteum at the same side	82	53	9	60.74 [52.50 - 68.98]
Adnexal rounded hyperechoic mass	63	57	8	52.50 [43.57 - 61.43]
Ring of fire sign	39	36	5	52.00 [40.69 - 63.31]
Yolk sac visible outside the uterine cavity	64	71	9	47.41 [38.99 - 55.83]
Adnexal mass as gestational sac with yolk sac	22	38	4	36.67 [24.48 - 48.86]
Caesarean section scar pregnancy peritrophoblastic blood flow	19	41	4	31.67 [19.90 - 43.44]
Intact endometrial midline echo	14	31	3	31.11 [17.58 - 44.64]
Tubal ring sign	45	120	11	27.27 [20.47 - 34.07]
Tubal ring without central identifying feature	24	66	6	26.67 [17.53 - 35.81]
Trophoblast visible outside the uterine cavity	110	325	29	25.29 [21.21 - 29.37]
Anterior distortion of uterus serosa	24	96	8	20.00 [12.84 - 27.16]
Smaller trophoblastic border distance to the anterior uterine serosa	33	162	13	16.92 [11.66 - 22.18]
Ectopic pregnancy wall more echogenic than corpus luteum wall	21	114	9	15.56 [9.45 - 21.67]
Fluid collection located centrally within the uterine cavity	4	41	2	13.33 [1.17 - 25.50]
Non intact endometrial midline echo	4	41	3	8.89 [0.57 - 17.21]
Gestational sac or trophoblast located at the level of internal cervical os	10	125	9	7.41 [2.99 - 11.83]
Gestational sac inside anterior myometrium and uterine cavity	1	44	3	2.22 [0.00 - 6.52]
Gestational sac located eccentricaly from uterine cavity	1	149	10	0.67 [0.00 - 1.98]
Total				40.35 [38.64 - 42.05]

## Discussion

We have developed an application ontology, a knowledge base and an application for the annotation of ultrasound images of ectopic pregnancy. This was the first attempt to build semantic resources in this domain. We discuss the significance of our findings, as well as the limitations and perspectives of this resource from the perspective of clinical application development.

### Significance

Using Sematic Web technologies [38] and ontologies [18], we successfully developed a comprehensive, unambiguous, shared and computable representation of the ectopic pregnancy ultrasound signs, for which existing resources were insufficient.

The ontology and the knowledge base received positive feedback from a panel of medical users (including mixed medical staff and sonographers). This preliminary evaluation demonstrates that they were able to identify morphological ultrasound features for a particular diagnosis and to associate them with pre-defined terms. The use of a large and diverse corpus as our source of vocabulary was critical for reaching a shared and fine-grained representation of the domain [20]. As expected, the signs described in the ontology are consistent with the most important signs for tubal pregnancy diagnosis identified in the recent meta-analysis by Richardson et al. [39].

The relevance of this application ontology is illustrated by a high precision rate of 83%, which reflects the proportion of correct sign annotations made by the observers. This result is especially encouraging at a time when we are considering using this knowledge base in a clinical decision support system.

The global proportion of agreement was 40.35%, which is satisfactory considering the number of images (33) and signs (26) involved. In comparison, a proportion of agreement of 50% was reported for the binary assessment of the abnormality of fetal heart rate in 20 cardiotocograms by 5 observers [37]. Interestingly, some signs with moderate proportions of agreement (e.g., “*tubal ring sign*”, *p*_*a =*_ 27.3% [20.5-34.1]_95%CI_), had good precision rates (e.g., precision = .77 for “*tubal ring sign*”). Moreover, in these images, a more general sign with a higher agreement was present (e.g., “*adnexal mass distinct from ovary*”, *p*_*a*_ = 79.4% [73.5-85.3]_95%CI_). This was an expected effect of sign suggestions in the application.

### Limitations

#### Limitations of the ontology

Many biomedical ontologies developed recently have used the basic formal ontology (BFO) [40] as their top-level ontology for interoperability with other OBO ontologies. Instead, we used our local core ontology for medicine (Ménélas), because interoperability with other projects in our institution was more important. Moreover, the use of a top-level ontology was not a primary requirement in the design of our application ontology. Similarly, we did not use the popular MIREOT [41] strategy for referencing external resources in our ectopic pregnancy ontology. Because it was crucial for this application ontology to ensure the stability of our application, we decided to restrict to a minimum the ontological commitment that comes with the reuse of external, evolving ontologies. However, we kept the mapping to reference resources, such as the FMA.

### Preliminary evaluation

In its current state, the application we developed only supports the annotation of clinical images, not the diagnosis of the conditions represented on these images. For our evaluation, most of the signs from the ontology used in annotations were tubal pregnancy signs, cesarean-section scar pregnancy signs and some signs that were not specific of a location. While sufficient for evaluating the precision and reproducibility of the annotations, this skewed dataset would be insufficient for the evaluation of a diagnostic system.

### Toward a clinical decision support system (CDSS) for ectopic pregnancy diagnosis

There is a need for CDSS in the domain of ultrasound signs for early pregnancy. Except in specialist centers, many women with ectopic pregnancy will not be diagnosed by transvaginal ultrasound at their first visit. However, adequate management necessitates detailed ultrasound differential diagnosis of the different early pregnancy complications [4, 42] which requires advanced training [43]. In practice, only some of the initial transvaginal scans are performed by experts, thus delaying the appropriate diagnosis and treatment, increasing adverse outcomes and also generating a significant number of visits [8, 44, 45]. In this context, a CDSS for early identification of relevant ectopic pregnancy signs will likely benefit non-expert operators. However, developing a CDSS for early pregnancy is challenging for several reasons. Potential users have heterogeneous expertise; there is no standard terminology describing the relevant ultrasound signs; and the quality of ultrasound images varies significantly among operators.

We consider this ectopic pregnancy image annotation application, with its underlying ontology and knowledge base, a step toward a clinical decision system for ectopic pregnancy diagnosis. Research in CDSS based on ontologies has demonstrated differential diagnosis assistance in Human Genetics [46] or in conventional Radiology [47].

The precision of the annotations derived from our knowledge base is promising for developing a CDSS for ectopic pregnancy ultrasound. The prospective evaluation of a clinical decision support system (CDSS) based on our knowledge base should demonstrate improvement in clinical care. For example, the expectation would be that, junior operators guided by the signs suggested by the system achieve a better analysis of ultrasound images, and therefore reach the correct diagnosis more often than without the system. A specific challenge for such clinical evaluation is its integration in the clinical workflow.

Finally, the knowledge base we developed could be extended from ectopic pregnancy to early pregnancy (i.e., including molar pregnancy, miscarriage and multiple pregnancy at early stages of development), and more generally to the next stages of fetal development (i.e., to represent ultrasound signs associated with fetal disorders).

## Conclusions

We have developed a new ectopic pregnancy knowledge base for the annotation of ultrasound images. The elements of this knowledge base (signs and types of ectopic pregnancy, anatomical structures involved and technical elements of imaging) are organized into an ontology. We have demonstrated the use of this knowledge base for the annotation of ultrasound images of ectopic pregnancy, with promising results from the perspective of clinical decision support system development. Other gynecological disorders and fetal abnormalities may benefit from our approach.

## Abbreviations

CDSS: Clinical decision support system
FMA: Foundational model of anatomy
NCBO: National center for biomedical ontology
NLP: Natural language processing
OBO: Open biomedical ontologies
OWL: Ontology web language
PMID: PubMed identification number
RadLex: Radiology lexicon
SKOS: Simple knowledge organization system
UCLH: University College London Hospital
UMLS: Unified Medical Language System
